# Changes in Organic Carbon Stock in Soil and Whole Tree Biomass in Afforested Areas in Latvia

**DOI:** 10.3390/plants12122264

**Published:** 2023-06-09

**Authors:** Guna Petaja, Arta Bārdule, Juris Zalmanis, Dagnija Lazdiņa, Mudrīte Daugaviete, Ilona Skranda, Zaiga Anna Zvaigzne, Dana Purviņa

**Affiliations:** 1 Latvian State Forest Research Institute “Silava”, Riga Street 111, LV-2169 Salaspils, Latvia; 2 Latvia University of Life Sciences and Technologies, Liela Street 2, LV-3001 Jelgava, Latvia

**Keywords:** afforestation, land use changes, soil organic carbon, tree biomass, bulk density

## Abstract

This study investigates the soil organic carbon (SOC) and whole tree biomass carbon (C), soil bulk density (BD) as well as changes in these parameters in afforested areas in Latvia. The study covered 24 research sites in afforested areas—juvenile forest stands dominated by Scots pine, Norway spruce and Silver birch. The initial measurements were conducted in 2012 and repeated in 2021. The results show that afforestation mostly leads to a general decrease in soil BD and SOC stock in 0–40 cm soil layer and an increase in C stock in tree biomass across afforested areas with various tree species, soil types, and former land uses. The physical and chemical properties of the soil could explain the differences in changes in soil BD and SOC caused by afforestation, as well as the impact of past land use may have persisted. When comparing the changes in SOC stock with the increase in C stock in tree biomass due to afforestation, taking into account the decrease in soil BD and the resulting elevation of soil surface level, the afforested areas at juvenile development stage can be considered a net C sink.

## 1. Introduction

Forests play an essential role in mitigating climate change since they act as carbon (C) sinks, sequestering atmospheric carbon dioxide (CO_2_) through photosynthesis and storing C in biomass and soils. In Europe, forests cover 43.5% of the land area. Latvia has the fourth largest forest area in the European Union (EU), corresponding to 56.2% of the total land area [1]. The forestry sector’s current management practices have been found to be highly effective in sequestering C, with EU forests annually sequestering CO_2_ equivalent to 7% of the EU’s total greenhouse gas (GHG) emissions [2]. Afforestation, or the establishment of forests in areas where they did not previously exist, has been identified as a potential means to increase C sequestration and mitigate climate change. In fact, afforestation projects in Europe have contributed significantly to the expansion of forest cover. In Latvia, according to the latest National GHG Inventory, there were 149.3 kha of land converted to the forest land in 2021 [3]. Afforestation is also considered a viable approach for improving soil quality through mitigating erosion, enhancing structure and porosity, reducing compaction, and promoting nutrient cycling [4,5].

The quantity of C stored in the soil is influenced by the equilibrium between the C inputs, such as litterfall and rhizodeposition, and C losses, primarily through decomposition of soil organic matter (SOM) [6,7,8,9,10]. Soil organic carbon (SOC) refers to C stored in the soil in the form of organic matter (OM). It plays a crucial role in maintaining soil structure, fertility, and water holding capacity [11]. The amount of organic C present in the top 100 cm of soil worldwide exceeds the amount found in the atmosphere, plants, or deeper soil C reserves [12]. In general, the process of afforestation has a beneficial impact on the balance of SOC in areas previously used for agriculture and with low levels of SOM. This is largely because tillage, which is a major cause of SOM depletion and reduced aggregation in soil, is prevented through afforestation [13,14]. The cessation of cultivation and the subsequent establishment of natural or perennial vegetation on agricultural land can result in the accumulation of SOC [12]. Over the short term, afforestation may lead to a reduction in SOC stocks, as the organic C primarily accumulates in the above-ground biomass. However, after several years, the accumulation of SOC reaches a level comparable to non-forested soils, and eventually, several decades after the afforestation process the SOC stock begins to increase more rapidly. Nevertheless, studies indicate that achieving the SOC levels found in natural forests requires a significant amount of time [15,16,17]. Furthermore, frequently significant translocation of SOC was observed within the soil profile, with a decrease in SOC and nitrogen (N) in deeper soil layer compensated by an increase in upper soil layer [18]. Results of a study carried out within the alpine regions of the European Alps shows that, while afforestation in some cases may not lead to significant alterations in the SOC stock, it does enhance the stability of SOM in the mineral soil [19].

Soil bulk density (BD), which refers to the mass of dry soil per unit volume, is another soil property affected by afforestation. Soil BD is not an inherent soil property because it can be influenced by external factors such as compaction, moisture content, and OM, and is not solely determined by the mineralogical and chemical composition of the soil [20]. Soil BD is considered a reliable indicator of soil compaction, which in turn impacts a range of soil properties and processes such as infiltration, rooting depth, soil porosity, aeration, available water capacity, nutrient availability, and soil microbial activity. These factors play a critical role in determining the overall productivity and health of the soil [21]. Soils with lower BD generally have better structure and provide a greater capacity to retain water, nutrients, and organic C compared to denser soils [20]. Soil BD measurements can enhance our comprehension of soil functions and provide insights into how land use impact soil organic C stocks. A recent study conducted in China showed that as the number of years of afforestation increased, there was a gradual decrease in BD [22].

By planting new trees, afforestation increases the amount of living biomass, which becomes a major C pool. Earlier observations showed that after afforestation of arable land around 30% of the total C is sequestered in the soil, with the remaining 70% being sequestered in the biomass; however, in general the amount of organic carbon (OC) stocks in the soil of forests is 1.5 times greater than the amount found in the biomass of trees [23,24]. As trees grow, changes in relatively simple metrics such as tree stem diameters can be measured to monitor increases in C storage in living above-ground tree biomass [25]. Afforestation also causes changes in below-ground living biomass (including coarse and fine tree roots) as well as debris (such as litter and dead roots). The estimation of changes in the below-ground pools is challenging to measure, which adds uncertainty to the overall potential of afforestation to influence OC [26]. To determine below-ground biomass from easily quantifiable stand variables, modeling approaches are frequently utilized instead of field measurements [27,28].

Despite the widely recognized significant increase in C stored in aboveground biomass following afforestation, the impact on SOC remains less clear, highlighting the importance of measuring changes in SOC stocks and identifying the underlying mechanisms, in addition to accurately estimating the C stored in tree biomass [29,30,31]. The aim of our study was to characterize soil BD, C concentration in soil, and SOC stock and its changes, as well as the C stock in tree biomass depending on tree species, soil type, and former land use. Studying SOC and soil BD after afforestation carries importance as it allows us to gain insights into C sequestration, assess soil health and fertility, provide guidance for sustainable land management practices, and inform policymaking and decision-making in the context of climate change mitigation and land use planning.

## 2. Results

### 2.1. Soil Bulk Density

**Figure 1:**
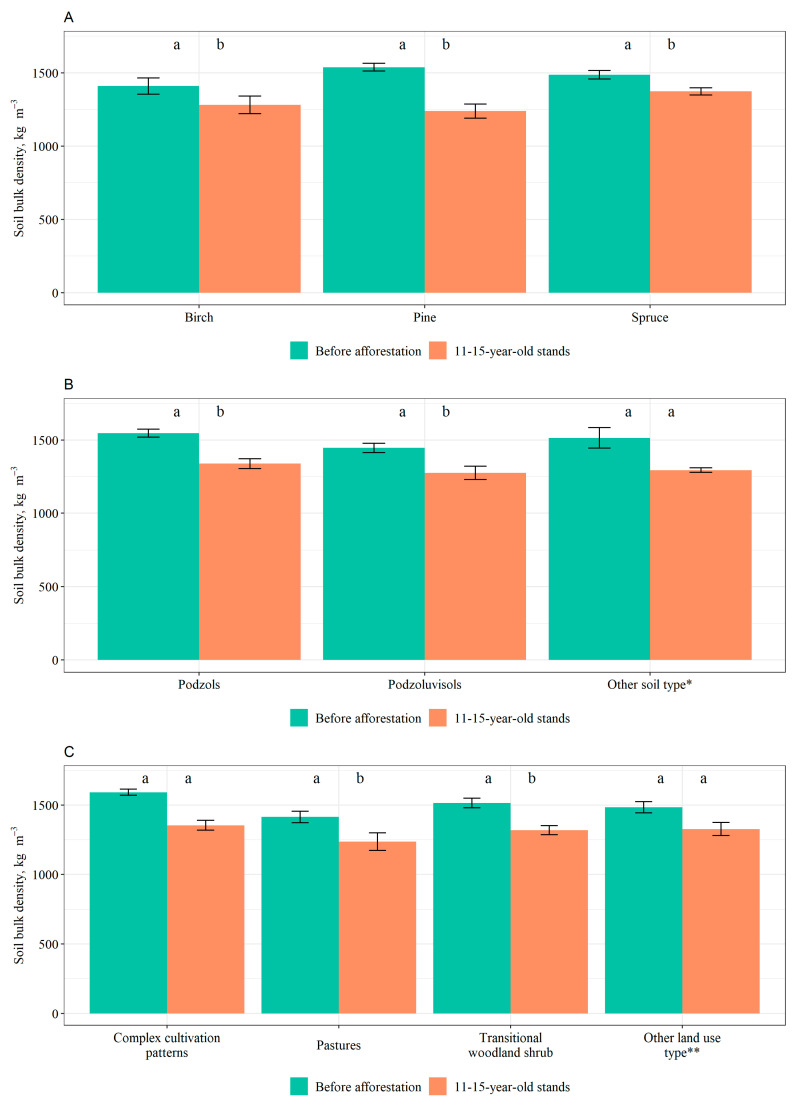
Average soil bulk density (BD) in 0–40 cm soil layer before afforestation and after afforestation in 11–15-year-old stands based on (**A**) dominant tree species, (**B**) soil type, and (**C**) former land use (mean values ± S.E. are shown). Different lowercase letters show statistically significant differences (*p* < 0.05, Wilcoxon signed-rank test) between the BD values before and after afforestation. * Includes Gleysols and Luvisols; ** Includes grassland, land mainly occupied by agriculture, with significant areas of natural vegetation, non-irrigated arable land.

Before afforestation, pine stands had the highest soil BD, followed by spruce stands, and then birch stands. However, after afforestation, there was a similar trend across stands of all tree species, with a general decrease in soil BD. After afforestation, the highest soil BD was measured in spruce stands, followed by birch stands, whereas pine stands had the most pronounced decrease in soil BD. Statistically significant differences in soil BD were observed for all tree species before and after afforestation: the *p*-value for pine was 0.004, for spruce it was 0.008, and for birch it was 0.016 (Figure 1A).

When examining changes in soil BD based on soil type, it was observed that Luvisols had the highest soil BD prior to afforestation, which decreased afterwards. Podzoluvisols had the lowest pre-afforestation soil BD, which also decreased following afforestation. In addition, Podzols and Gleysols experienced a decrease in soil BD after afforestation. Statistically significant differences for soil BD before and after afforestation were found for Podzols (*p* = 0.016) and Podzoluvisols (*p* < 0.001) (Figure 1B).

Also, when analyzing changes in soil BD after afforestation based on former land use, in all cases a decrease in soil BD was observed. The highest soil BD before afforestation was observed in areas where the former land use was complex cultivation patterns, whereas after afforestation the highest soil BD was observed in former non-irrigated arable land. The lowest soil BD before and after afforestation was observed in former pastures. Statistically significant differences in soil BD before and after afforestation were found for pastures (*p* = 0.004) and transitional woodland shrub (*p* = 0.031), whereas no significant differences were found between different land uses (Figure 1C).

No significant correlations were found between changes in soil BD due to afforestation and both other soil chemical parameters and tree stand characteristics.

### 2.2. Soil Organic Carbon

#### 2.2.1. Soil Organic Carbon Concentration

**Figure 2:**
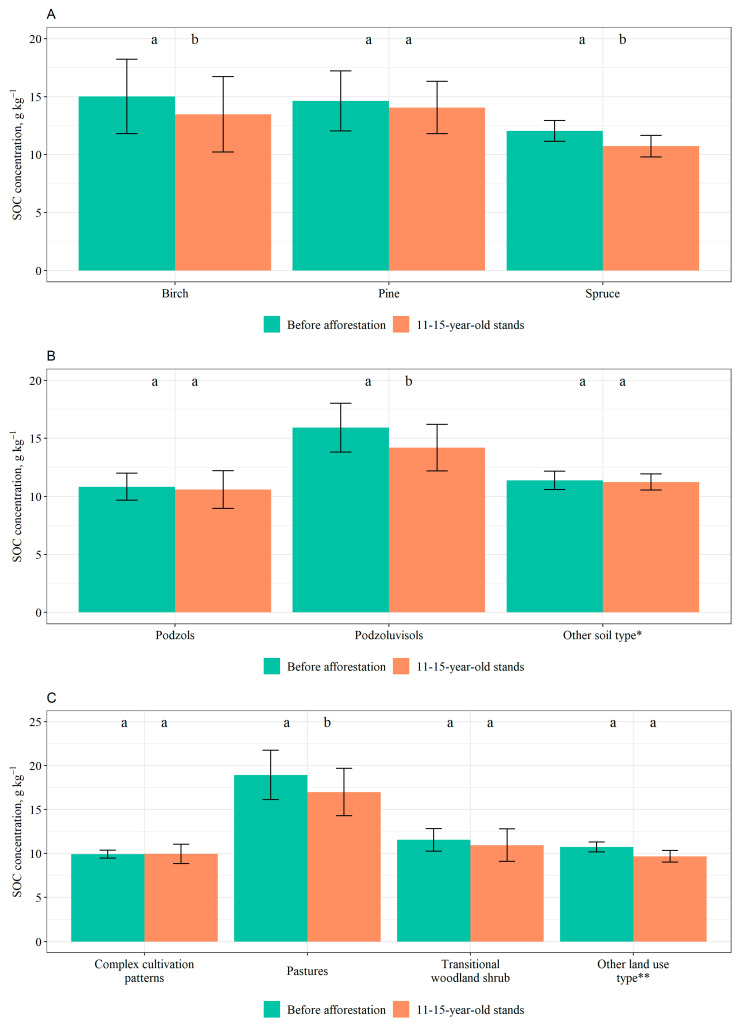
Average soil organic carbon (SOC) concentration in 0–40 cm soil layer before afforestation and after afforestation in 11–15-year-old stands based on (**A**) dominant tree species, (**B**) soil type, and (**C**) former land use (mean values ± S.E. are shown). Different lowercase letters show statistically significant differences (*p* < 0.05, Wilcoxon signed-rank test) between the SOC values before and after afforestation. * Includes Gleysols and Luvisols; ** Includes grassland, land mainly occupied by agriculture, with significant areas of natural vegetation, non-irrigated arable land.

Areas that were later afforested with birch had slightly higher SOC concentrations before afforestation than those afforested with pine and spruce. However, after afforestation, the highest SOC concentration was found in pine stands and there was a decrease in the average SOC concentration in 0–40 cm soil layer in stands of all the tree species compared to before afforestation. The differences in SOC concentrations before and after afforestation were statistically significant for birch (*p* = 0.016) and spruce stands (*p* = 0.008) (Figure 2A).

When considering SOC concentrations in 0–40 cm soil layer based on soil types, Podzoluvisols had the highest concentration before and after afforestation, compared to other types. A slight decrease in SOC concentration is observed for all the soil types after afforestation except for Luvisols, where a slight increase is observed. The difference in SOC concentration before and after afforestation for Podzoluvisols was statistically significant (*p* < 0.001) (Figure 2B).

When considering SOC concentrations in 0–40 cm soil layer based on former land uses, considerably higher concentrations before and after afforestation were found in pastures, compared to other land uses. A slight increase was observed for complex cultivation patterns, and land mainly occupied by agriculture with significant areas of natural vegetation, whereas a slight decrease was observed for other land uses. The difference in SOC concentration in 0–40 cm soil layer in pastures before and after afforestation was statistically significant (*p*-value = 0.004) (Figure 2C).

#### 2.2.2. Soil Organic Carbon Stock

**Figure 3:**
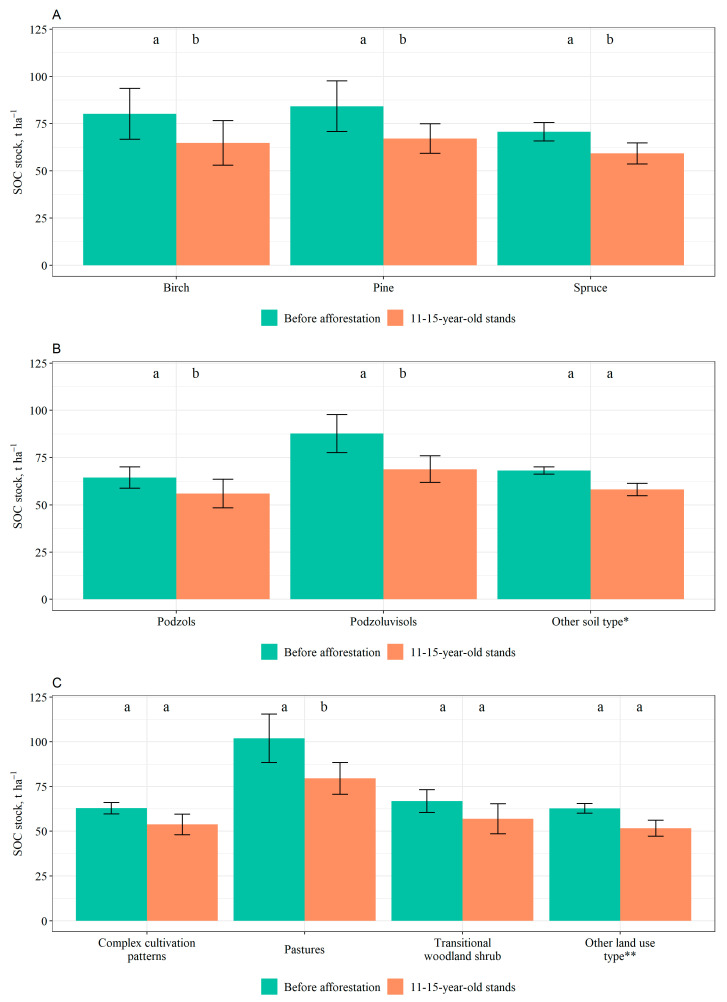
Soil organic carbon (SOC) stock in 0–40 cm soil layer before afforestation and after afforestation in 11–15-year-old stands based on (**A**) dominant tree species, (**B**) soil type, and (**C**) former land use (mean values ± S.E. are shown). Different lowercase letters show statistically significant differences (*p* < 0.05, Wilcoxon signed-rank test) between the SOC stock values before and after afforestation. * Includes Gleysols and Luvisols; ** Includes grassland, land mainly occupied by agriculture, with significant areas of natural vegetation, non-irrigated arable land.

After afforestation, there was a tendency for a decrease in the average SOC stock in 0–40 cm soil layer in stands of all the tree species, compared to before afforestation. The SOC stock before and after afforestation was the highest in pine stands, followed by birch and spruce stands, respectively. The differences in SOC stocks in 0–40 cm layer before and after afforestation for each species before and after afforestation were statistically significant: the *p*-value was 0.012 for pine, 0.008 for spruce, and 0.016 for birch (Figure 3A).

When considering SOC stock in 0–40 cm soil layer based on soil types, the largest stock before and after afforestation was found in Podzoluvisols, but the lowest before afforestation was in Gleysols and after afforestation was in Podzols. The differences in SOC stock in 0–40 cm soil layer before and after afforestation in Podzols (*p*-value = 0.047) and Podzoluvisols (*p*-value < 0.001) were statistically significant (Figure 3B). 

In addition, when analyzing the differences in SOC stocks in 0–40 cm layer before and after afforestation based on former land use, the largest stock was found in pastures both before and after afforestation. For the rest of the land uses the SOC stocks were considerably lower. The lowest SOC stock was found in grassland both before and after afforestation. A decrease in SOC stock after afforestation was observed for all the land use types. The difference in SOC stock before and after afforestation in pastures was statistically significant (*p* = 0.004) (Figure 3C).

### 2.3. Tree Biomass

**Figure 4:**
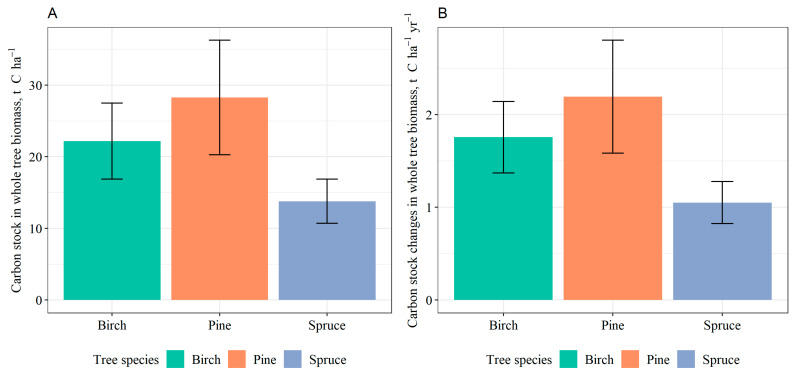
Total carbon stock (**A**) and annual stock changes (**B**) in whole tree biomass in afforested areas (11–15 years old stands) based on dominant tree species (mean values ± S.E. are shown).

Areas afforested with pine had the highest C stock in whole-tree biomass, followed by those afforested with birch and spruce (Figure 4A). In addition, changes in C stock in biomass per year were the highest in pine stands (2.19 ± 0.61 t C ha^−1^ yr^−1^), followed by birch (1.76 ± 0.39 t C ha^−1^ yr^−1^) and spruce (1.05 ± 0.23 t C ha^−1^ yr^−1^) (Figure 4B). No statistically significant differences were observed in terms of C in the overall tree biomass among different dominant tree species in the afforested areas. Additionally, no significant differences among the species were found regarding the annual changes in C stock.

### 2.4. Total Impact of Afforestation on C Stock in Soil and Tree Biomass 

**Figure 5:**
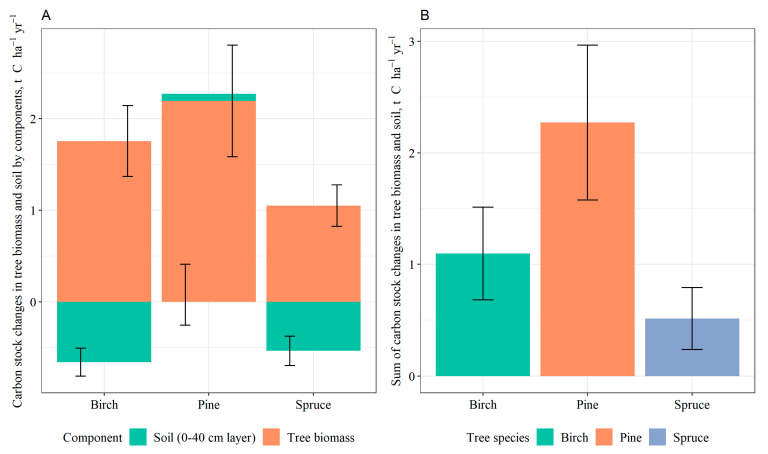
Annual C stock changes in soil (0–40 cm soil layer) and whole tree biomass in afforested areas (juvenile stands) based on dominant tree species (mean values ± S.E. are shown). (**A**) Carbon stock changes in tree biomass and soil by components. (**B**) Sum of carbon stock changes in tree biomass and soil.

Average annual SOC stock changes in 0–40 cm soil layer (calculated taking into account decrease in soil BD and thus elevation of soil surface level) in juvenile stands were −0.66 ± 0.15 t C ha^−1^ yr^−1^ in birch stands, −0.54 ± 0.16 t C ha^−1^ yr^−1^ in spruce stands, and 0.08 ± 0.33 t C ha^−1^ yr^−1^ in pine stands (Figure 5A). Average annual C stock changes in whole tree biomass were 1.76 ± 0.39 t C ha^−1^ yr^−1^ in birch stands, 1.05 ± 0.23 t C ha^−1^ yr^−1^ in spruce stands, and 2.19 ± 0.61 t C ha^−1^ yr^−1^ in pine stands. The decreases in SOC stock in birch and spruce stands were compensated by increase in C stock in tree biomass resulting in total C stock increase in afforested areas. The sum of carbon stock changes (increase) in tree biomass and soil (0–40 cm soil layer) was 1.10 ± 0.42 t C ha^−1^ yr^−1^ in birch stands, 0.51 ± 0.28 t C ha^−1^ yr^−1^ in spruce stands, and 2.27 ± 0.69 t C ha^−1^ yr^−1^ in pine stands (Figure 5B). 

## 3. Discussion

### 3.1. Soil Bulk Density

The results of our study indicate a general decrease in soil BD in 0–40 cm soil layer over a nine-year period in afforested areas at juvenile developmental stages compared to the pre-afforestation period. Previous studies show that the identity of a species plays a significant role in shaping soil properties, particularly in the uppermost layer of forest soil, although the information on this topic is scarce. The role of tree species has been predominantly discussed in relation to SOC [32,33]. Our findings did not reveal any significant differences in soil BD in 0–40 cm soil layer between the forest stands of different tree species, while the difference in soil BD in 0–40 cm soil layer before and after afforestation is significant for all the tree species. No significant differences in soil BD were found between the soil types; however, significant differences before and after afforestation were found for Podzols and Podzoluvisols. Physical and chemical characteristics could be the reason why Podzols and Podzoluvisols react to afforestation at different rates than other soil types. Podzols and Podzoluvisols are both characterized by low OM content in the leached surface, high acidity, and low clay content, while Luvisols are less acidic. Both Luvisols and Gleysols are comparatively fertile and have higher clay content. In addition, no significant differences in soil BD in 0–40 cm soil layer were found between the land uses, but there are significant differences in soil BD in 0–40 cm soil layer before and after afforestation for pastures and transitional woodland-shrub. Previous uses for agriculture in case of complex cultivation patterns, grassland, and land mainly occupied by agriculture with significant areas of natural vegetation may have a longer-lasting effect. As soil BD for complex cultivation patterns was the highest before afforestation, it may take more time for it to become significantly lower compared with the pre-afforestation period.

The utilization of machines in agricultural practices as well as animal trampling leads to soil compaction, increased soil BD, and physical deterioration [34,35,36]. Numerous studies have reported a decrease in soil BD following afforestation, which positively affected soil quality. In a study carried out in larch (*Larix gmelinii*) plantations in China, a significant decrease in soil BD was found in the upper layer of soil (0–20 cm) 25 years after afforestation of farmland as well as in a chronosequence plot series, whereas no significant changes were observed in the deeper layers [37]. In another study carried out in China, the mean soil BD in the 0–1.0 m soil profile was lower in various forested treatments (16–40-year-old) than both the abandoned and most of the cultivated treatments [4]. In addition, a study carried out in the Czech Republic showed that afforestation of arable land, where tree species included pine, spruce, and birch, have reduced soil BD. However, this study observed a significant and persistent influence from the previous land use [5]. In a study carried out in Spain, infiltration rates were significantly higher in the recently afforested pine area as compared to the land without trees (shrubs and grasslands), and marginally higher than the infiltration rate in the broadleaf holm oak (*Quercus ilex*) area [38]. A study carried out in Finland, which also included Gleysols and Podzols, showed that soil properties of afforested areas considerably differ from natural forests on mineral soils [39]. In another study carried out in Finland where the sites were afforested either 10 or 60–70 years ago with Norway spruce, soil characteristics were examined. The afforestations that were 10 years old exhibited a tendency for lower soil BD of mineral soil and higher OM content compared to sites that were continuously forested [40]. In addition, in this case the effect of former land use had been long lasting. In a study carried out in Lithuania the average bulk density of fine (<2 mm) soil in the 0–30 cm layer was found to be higher in croplands compared to afforested areas (20–30 years after afforestation) and grasslands [17].

### 3.2. Soil Organic Carbon Concentration and Stock

In addition, SOC concentrations and stocks in 0–40 cm soil layer exhibited a general decrease after afforestation. Studies show that tree species planted significantly influences SOC changes following afforestation. This is because forest litter (both aboveground and belowground) serves as a substantial contributor to the input of SOM [41,42]. Different tree species exhibit variations not only in their litter production but also in the rates at which their litter decomposes, which contribute to the dynamics of SOC stock changes. The decomposition of broadleaved litter is faster compared to coniferous litter due to its lower lignin content. The rate at which litter decomposes is also influenced by the composition and arrangement of the foliage [41]. Decomposition of litter from evergreen trees typically occurs at a slower rate compared to litter from deciduous trees [42]. In our study changes in SOC were not species-specific, as there were no significant differences between pine, spruce, and birch stands. When comparing the SOC stock in the 0–40 cm soil layer before and after afforestation, significant differences were observed for all tree species, disregarding changes in soil BD due to afforestation and the resulting elevation of the soil surface level. Additionally, in the case of birch and spruce stands, there were significant differences in SOC concentration within the 0–40 cm soil layer. Areas that were later afforested with birch had the highest SOC concentrations in 0–40 cm soil layer before afforestation, whereas after afforestation, the highest SOC concentration was found in pine stands. In case of SOC stock in 0–40 cm soil layer, the highest values were found for pine stands before and after afforestation; however, pine stands also had the largest reduction in SOC stock compared to SOC stock in 0–40 cm soil layer without taking into account changes (decreases) in soil BD due to afforestation. Results of a meta-analysis indicate that afforestation of cropland and grassland with deciduous broadleaved trees resulted in the most consistent and significant increase in SOC stock compared to other tree species. Conversely, afforestation with conifer trees showed the lowest rate of SOC stock change, which contradicted our results [43]. Another meta-analysis, where former land uses were mostly grasslands, pastures, and abandoned agricultural lands, showed a similar trend [44]. According to this analysis [44] afforestation resulted in a decrease in SOC and the effect was the greatest for pine plantations, decreasing soil C stocks by 15%. This finding is similar to our results. Our results could potentially be explained by differences in decomposition rates of litter among the tree species.

In addition, across different soil types there was a general decrease in SOC concentration and stock in 0–40 cm soil layer after afforestation when comparing SOC stock without taking into account changes (decrease) in soil BD due to afforestation. Statistically significant differences were found for Podzols and Podzoluvisols regarding SOC stock and for Podzoluvisols regarding SOC concentration. In addition, in this case soil physical characteristics may play a role. Among the studied soil types Podzols have the lowest clay content, followed by Podzoluvisols. It has been concluded that soils rich in clay have a better capacity to accumulate SOC [45,46]. In a study carried out in Lithuania the soil type and the composition of stand species (deciduous versus coniferous) did not exhibit significant variations in SOC stocks within afforested sites [17].

Across different former land uses, complex cultivation patterns exhibited a slight increase in SOC concentration, whereas for the rest of the land uses a decrease was observed in both SOC stock in 0–40 cm soil layer, when comparing SOC stock without taking into account changes (decreases) in soil BD and thus elevation of soil surface level and SOC concentration. In general, lands used for agriculture showed less prominent decrease in SOC, comparing with pastures. Pastures exhibited the sharpest decrease and a significant difference in SOC stock before and after afforestation. In a study carried out in Norway, where the soil type was Podzoluvisols, it was found that even 50 years after afforestation of a pasture, there was no increase in SOC. It was concluded that the previous cultivation practices still had an impact on the current state of soil productivity and organic matter dynamics [47]. According to a review study on the temperate climatic zone, where a long-term effect of afforestation was modelled, afforesting grasslands did not result in a soil organic carbon (SOC) sink, and in 75% of cases, SOC losses were observed even after a century [48]. A recent review study shows that SOC after afforestation is significantly affected by previous land use at a depth of 60 cm on former barren land and cropland. However, no significant changes in SOC stock were observed following afforestation on grassland [49].

Generally, different conclusions have been made from previous studies regarding how afforestation affects SOC. Some studies have found no change in SOC stocks after afforestation. In a study carried out in New Zealand, where soil properties under grassland were compared to those of first-rotation pine forest on mineral soils, the OC pool in forests was lower than in grasslands, but differences were not statistically significant. In addition, this difference was measured only in the 0–0.1 m layer, whereas in the 0–0.3 m layer the C pool was slightly higher for forests; however, the difference was not statistically significant also in this case [50]. At the Kellogg Biological Station in the USA, Michigan, three land-use treatments were compared with a neighboring deciduous forest as part of a long-term ecological research project. These treatments included conventionally tilled cropland and a former cropland afforested with poplar. Total soil C was found to be similar between the cropland and the poplar afforested system when examining the 0–50 cm soil layer. The study put forward a hypothesis that it may take about 10–40 years before a noticeable rise in subsurface C stocks can be observed in afforested systems [15]. In a study carried out in south-east Poland, where former arable land was afforested with Scots pine, it was found that the OC content in the 0–20 layer was initially lower in the afforested site, but it ultimately recovered to reach a level that is typical of arable soils [16]. In a study carried out in Denmark, where arable soils were afforested with Norway spruce and oak, it was found that the C concentration and storage for 0–25 cm decreased in the short term [51]. The findings of a meta-analysis on C accumulation in agricultural soils after afforestation suggest that the factors that primarily contribute to the restoration of SOC stocks following afforestation are the previous land use, the tree species selected for planting, the soil clay content, and the extent of preplanting disturbance [45].

Some studies have found an increase in SOC stocks. According to a review on soil C after land use changes in temperate, subtropical, and tropical regions a decrease in C for cultivated fields to pine plantation and an increase for long term agriculture and 10-year-old crop fields to secondary forest and mahogany plantation and cultivated field to Eucalyptus plantation were found. The findings of another review study revealed that soil C stocks increase after land use changes from crop to plantation (+18%) and from crop to secondary forest (+53%), whereas it declines when land use changes from pasture to plantation (−10%) [52]. In a study carried out in China, mangrove afforestation in 20 years increased SOC stock by 61.29% without significant variation among different mangrove species [53]. The findings of another recent study demonstrated that afforestation of farmland in Southwest China led to significant increases in the total SOC content and its fractions [54]. A study conducted in Lithuania, focusing on the afforestation of cropland and grassland with nutrient-poor Arenosols and nutrient-rich Luvisols respectively, revealed that SOC exhibited a greater increase in Arenosols [17]. Trees in boreal climate zones exhibit a decreased growth rate, produce less litterfall, and experience slower decomposition of litter, which collectively result in a reduced input of SOC [43,45]. 

As forest stands become more productive, the SOM input gradually increases and the soils shift from serving as a C source to a C sink. As a result, soil C can eventually recover to its pre-afforestation levels and, in certain circumstances, even exceed them. [45,55,56,57,58,59].

### 3.3. Carbon Stock in Tree Biomass

Areas afforested with pine had the highest C stock in whole-tree biomass, followed by those afforested with birch and spruce. In addition, changes in C stock in biomass per year were the highest in pine stands, followed by birch and spruce. Comparing the decrease in SOC and the increase in biomass OC, the equal or even greater amount of C than lost in soil was stored in tree biomass or litter. A majority of studies suggest that the SOC losses are typically modest in comparison to the accumulation of C in tree biomass [57,60]. However, these studies are looking at the global scale, while the climatic zone should be taken into account. The slow growth of coniferous trees in boreal climates means that even minor losses of SOC can cancel out the C sequestered by these trees [47].

In a study carried out in China it was found that coniferous forests had a higher C storage in living biomass than broadleaf stands [6]. In a study carried out in subalpine ecosystems of the Swiss National Park, it was found that aboveground C stocks were significantly higher in an early successional forest, comparing with short-grass pastures, whereas belowground stocks did not differ significantly [61]. 

It is possible that the amount of OC stock in biomass is slightly higher than our estimates, considering the OC in biomass of ground vegetation. Ground vegetation, such as herbs, shrubs, and grasses, is another significant component of forest ecosystem, but it is often excluded from C budget analyses. Research conducted in pine and spruce upland forests in Finland has shown that ground vegetation accounted for approximately 4–13% of the C stock, as reported by Mälkönen and Havas and Kubin [62,63]. However, other studies have found that the proportion of C stored in ground vegetation is lower, around 1–2%, as reported by Lakida et al. and Pussinen et al. [64,65]. Accurately estimating C input through ground vegetation requires an investigation of site attributes that can be used to predict ground vegetation biomass [66,67]. For forests in Latvia, developing country-specific equations to calculate ground vegetation biomass is also necessary [68]. 

## 4. Materials and Methods

### 4.1. Research Sites

**Figure 6:**
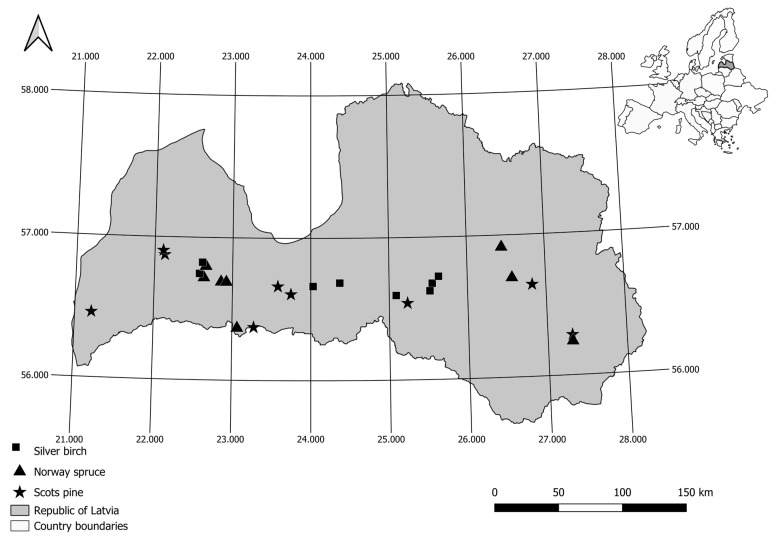
Location of the study sites (afforested areas) in Latvia. Different dominant tree species of each site are shown with different shapes.

The study was conducted in 24 research sites (afforested areas) across the southern part of Latvia (Figure 6). The dominant tree species in studied afforested areas were Scots pine (*Pinus sylvestris* L.), Norway spruce (*Picea abies* (L.) H.Karst.), and Silver birch (*Betula pendula* Roth). The initial soil and tree measurement was conducted during the spring season of 2012, from February to April, describing the conditions (soil physico-chemical parameters) before afforestation. Subsequently, after a gap of 9 years, measurements were repeated during the spring season of 2021, from March to April. 

The climatic standard norm of the annual average air temperature (1991–2020) in Latvia is +6.8 °C. Annual precipitation in Latvia is 685.6 mm. During the study period (2012–2021), the mean annual precipitation in Latvia ranged from 473 to 832 mm, while the mean annual air temperature ranged from +6.1 to +8.8 °C [69].

Measurement plots, with an area of 60 × 60 m, were established in each research site (afforested area), at a minimum distance of 20 m from the neighboring stands, roads, or areas with other land use. Within this compartment, round measurement plots with a total area of 500 m^2^ were established in the center. To mark the center of the plot, a pole and metal stick were inserted into the soil, which can be easily detected later with a metal detector. All trees within the plot were counted and their height was measured. In addition, forest stand data were obtained from the State forest service’s stand-wise forest inventory [70]. Initial land use was determined using Corine land cover year 2000 map. 

### 4.2. Soil Sampling and Analyses 

Soil samples were collected from each plot at three different depths (0–10 cm, 10–20 cm, and 20–40 cm) using a soil auger in both 2012 and 2021. During the first measurement period, soil samples were taken 3–5 m north and south from the border of the measurement plot. In the second measurement period, soil samples were taken 3–5 m east and west from the measurement plot. Soil sample for BD was taken from the middle of each selected soil layer, while soil samples for determination of C concentration were taken from the entire depth of the soil. The methods used for soil sample preparation and analysis are summarized in Table 1, and they fully comply with the requirements of ICP Forests [71]. The soil type was identified by analyzing the soil and assigning the results to the FAO major soil groupings, using the methodology proposed by Kārkliņš [72].

The OC concentration in soil, soil BD, and fine fraction (particles with a diameter below 2 mm) were used for the calculation of SOC stock in 0–40 cm soil layer. It was assumed that the coarse fraction of soil does not contain organic C. Changes in SOC stocks in 0–40 cm soil layer due to afforestation during the study period (Figure 5) were calculated by taking into account changes (decrease) in soil BD and thus elevation of soil surface level (thickness of the soil layer in the first measurement was proportionally reduced).

### 4.3. Tree Biomass 

Biomass of trees (including roots with at least 2 mm in diameter) in afforested areas was calculated using above- and below-ground biomass equations elaborated in previous studies implemented in Latvia [73,74]. The calculations were performed at a single tree level and then extrapolated to a plot level.

(1) $$y=a*exp\left(\frac{-1}{2}*{\left(\frac{H-b}{c}\right)}^{2}+{\left(\frac{D-d}{e}\right)}^{2}\right)$$

where:

*y*—dry biomass fraction, kg
*H*—stem height, m
*D*—diameter at breast height, cm
*a*, *b*, *c*, *d*, *e*—equation parameters

The parameters of the biomass equation are given in Table 2. 

In order to calculate C stock in tree biomass, values (C content in tree biomass by tree parts and species) given in Table 3 were used.

## 5. Conclusions

The findings of our study suggest that afforestation has a significant impact on reducing soil BD. This impact is not species-specific. The physical and chemical properties of the soil may be the reason why the reduction in soil BD is significant for Podzols and Podzoluvisols but not for the other soil types examined when subjected to afforestation. Previous land use may have had a lasting effect on soil BD, leading to a significant response of afforestation for pastures and transitional woodland shrub, but not for complex cultivation patterns, land mainly occupied by agriculture, land with significant areas of natural vegetation, and non-irrigated arable land. 

Also, SOC mostly shows a decrease in the early years after afforestation, which is a previously documented response in the short term. Similarly to soil BD, in case of SOC concentration in 0–40 cm soil layer, significant differences comparing with the pre-afforestation period were found for birch and spruce stands, Podzoluvisols and pastures, whereas in case of SOC stock differences were across stands of all the tree species, Podzols and Podzoluvisols, and pastures. 

When comparing the changes in SOC stock due to afforestation, taking into account decease in soil BD and thus elevation of soil surface level, with the increase in C stock in tree biomass, afforested areas at juvenile development stage were C sinks (average annual sum of carbon stock changes in tree biomass and soil (0–40 cm soil layer) ranged from 0.51 ± 0.28 t C ha^−1^ yr^−1^ in spruce stands to 2.27 ± 0.69 t C ha^−1^ yr^−1^ in pine stands).

This study offers new insights into the soil conditions of afforested areas in Latvia. The SOC and tree biomass C values obtained from this study are valuable for improving the national greenhouse gas inventory, specifically the Land Use, Land-Use Change, and Forestry (LULUCF) sector. The findings of our study are valuable for policymakers and land managers who are dedicated to climate change mitigation in forested regions. Additionally, they provide useful information for individuals interested in improving soil quality after agricultural and grazing activities. The findings of our study can be applicable not only in Latvia but also in other countries within the hemiboreal zone.

However, in order to evaluate the effect of afforestation on total C stock changes, long-term observations are desirable. In colder climates soil properties change slowly, therefore it may take decades after afforestation for the amount of SOC to increase significantly. 

## Figures and Tables

**Table 1:** Soil analysis methods and analytical instruments used.

Parameter	Method ISO Reference	Principle	Method Description (Analytical Equipment)
Soil moisture	LVS ISO 11465:2006	Gravimetry	Air-dried and treated soil samples are dried at 105 °C (Sartorius AX224)
Content of carbonates	LVS EN ISO 10693:2014	Volumetry	Samples are treated with 4M HCl solution and volume of produced CO_2_ is measured (Eijkelkamp calcimeter)
Content of total carbon	LVS ISO 10694:2006 ISO 15178:2000 LVS ISO 13878:1998	Dry incineration	Dry incineration at 950 °C temperature (Elementar EL Cube)
Soil preparation	LVS ISO 11464:2006	Drying and sieving	Drying at 40 °C temperature and separation of fine fraction for chemical analyses
Bulk density	LVS ISO 11272:2017	Soil cylinder method	Dry mass (dried at 105 °C) of 100 cm^3^ of undisturbed soil samples

**Table 2:** Parameters of Gaussian function equation for above- and below-ground biomass of the studied tree species [74].

Coefficient	Pine		Spruce		Birch	
	Above-Ground Biomass	Below-Ground Biomass	Above-Ground Biomass	Below-Ground Biomass	Above-Ground Biomass	Below-Ground Biomass
*a*	2273.58	553.59	1902.43	29,714.37	1261.84	338.25
*b*	62.32	21.77	33.31	131.71	43.41	17.87
*c*	31.65	10.75	15.62	31.92	20.89	16.86
*d*	53.86	51.97	63.39	25.56	44.34	44.45
*e*	18.69	14.01	22.3	11.6	15.15	12.88

**Table 3:** Carbon content in above- and below-ground biomass of the studied tree species [74].

Tree Species	Carbon Content, g C kg^−1^		Uncertainty, g C kg^−1^	
	Above-Ground Biomass	Below-Ground Biomass	Above-Ground Biomass	Below-Ground Biomass
Birch	520.01	519.85	0.22	1.22
Spruce	525.59	526.07	1.15	0.62
Pine	531.17	548.08	0.43	0.97

## Data Availability

Data supporting reported results can be acquired by contacting the corresponding author.
